# Targeting Type II Toxin–Antitoxin Systems as Antibacterial Strategies

**DOI:** 10.3390/toxins12090568

**Published:** 2020-09-04

**Authors:** Marcin Równicki, Robert Lasek, Joanna Trylska, Dariusz Bartosik

**Affiliations:** 1 Centre of New Technologies, University of Warsaw, ul. S. Banacha 2c, 02-097 Warsaw, Poland; joanna@cent.uw.edu.pl; 2 Department of Bacterial Genetics, Institute of Microbiology, Faculty of Biology, University of Warsaw, Miecznikowa 1, 02-096 Warsaw, Poland; lasek@biol.uw.edu.pl

**Keywords:** toxin–antitoxin systems, toxin activation, antibacterial agents, bacterial persistence

## Abstract

The identification of novel targets for antimicrobial agents is crucial for combating infectious diseases caused by evolving bacterial pathogens. Components of bacterial toxin–antitoxin (TA) systems have been recognized as promising therapeutic targets. These widespread genetic modules are usually composed of two genes that encode a toxic protein targeting an essential cellular process and an antitoxin that counteracts the activity of the toxin. Uncontrolled toxin expression may elicit a bactericidal effect, so they may be considered “intracellular molecular bombs” that can lead to elimination of their host cells. Based on the molecular nature of antitoxins and their mode of interaction with toxins, TA systems have been classified into six groups. The most prevalent are type II TA systems. Due to their ubiquity among clinical isolates of pathogenic bacteria and the essential processes targeted, they are promising candidates for the development of novel antimicrobial strategies. In this review, we describe the distribution of type II TA systems in clinically relevant human pathogens, examine how these systems could be developed as the targets for novel antibacterials, and discuss possible undesirable effects of such therapeutic intervention, such as the induction of persister cells, biofilm formation and toxicity to eukaryotic cells.

## 1. Introduction

The emergence of antibiotic-resistant bacteria is a critical issue in modern medicine [1]. Considerable efforts are being made to identify novel antimicrobials to combat infectious diseases caused by evolving multi-resistant pathogens. Bacterial toxin–antitoxin (TA) systems represent promising targets for such compounds. These small genetic modules encode two components: a stable toxin (always a protein) that recognizes a specific cellular target, and a labile antitoxin (protein or RNA molecule), produced in excess, that counteracts the activity of the toxin [2]. TAs have been classified into several groups based on the molecular nature of the antitoxin and their mode of interaction with their cognate toxin. The most prevalent and most extensively studied are type II TA systems, encoding proteic antitoxins which neutralize the toxin by forming TA complexes under optimal growth conditions [3,4]. These two-gene *loci* are organized in operons, whose expression is tightly regulated by both the antitoxin and toxin–antitoxin complexes [5,6]. Degradation of the antitoxin by cellular proteases liberates the toxin, which elicits a bacteriostatic or bactericidal effect [7,8,9,10].

TA *loci* were initially identified within bacterial plasmids, where they function as post-segregational cell killing systems (PSK), providing stable maintenance for their carrier replicon in a bacterial population due to the elimination of plasmid-less cells [11]. A surprising observation was that TA systems are also highly abundant and widespread in the chromosomes of free-living bacteria (both Gram-negative and Gram-positive), as well as in archaea [12]. More detailed studies have revealed that these TAs may be involved in important biological processes, such as (i) the stringent response, helping cells to survive stressful conditions by limiting various metabolic activities [13], (ii) programmed cell death [14] and (iii) biofilm formation [15]. In the light of these observations, it seems likely that chromosomal TA *loci* have been transferred to extrachromosomal replicons and adopted as plasmid stabilization systems.

The toxins of TA systems target essential cellular processes of bacteria; therefore, they may be considered intracellular “molecular time bombs”, which are activated under certain environmental conditions [16]. Compounds that can artificially activate TA toxins may form a new class of antimicrobials that could represent an alternative to antibiotics [17]. Type II TA systems are ubiquitous in bacteria [18], and their mechanisms are fairly well characterized [18], so they would appear to be excellent candidates for testing the merits of this idea.

The potential use of TA systems in combatting bacterial infections has been examined in several valuable reviews [19,20,21,22]. Here, we describe the most promising antibacterial strategies employing type II TA systems and discuss possible undesirable effects of their application, such as the induction of persister cells, biofilm formation and toxicity to eukaryotic cells. In addition, the distribution of type II TA systems among clinically relevant human pathogens is described, which may help to identify appropriate TA candidates for fighting particular infections.

## 2. Type II TA Systems in Pathogenic Bacteria

**Figure 1:**
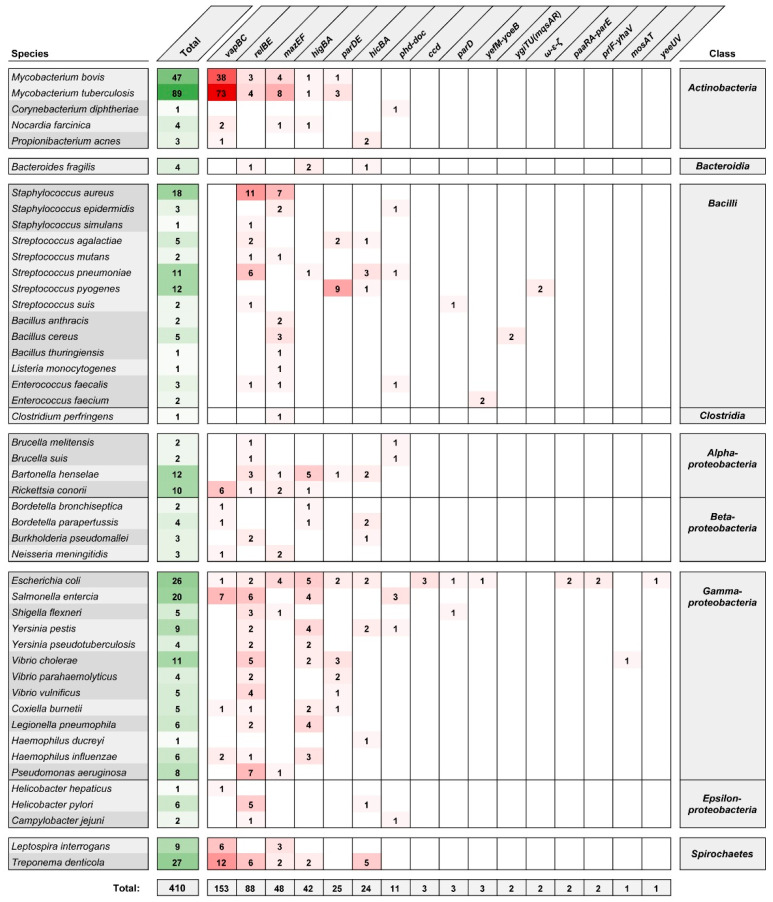
Type II toxin–antitoxin (TA) systems identified in human pathogenic bacteria according to the toxin family classification system [25,26] as collected in the Toxin–Antitoxin Database (TADB) 2.0 [24]. The number given for an individual species is the sum of the values for all strains of a given taxon. This list was manually curated to represent strains and/or species frequently associated with infections in humans (including opportunistic pathogens).

Since 2011, the studies of TA systems have been assisted by the Toxin–Antitoxin Database (TADB) [23], updated to version 2.0 in 2017 [24]. Currently, TADB 2.0 lists 6194 type II TA *loci* [24] categorized, where possible, in two partially interdependent ways. The first classification scheme uses a system based on the structural and functional characteristics of the toxin proteins [25,26]. There are 11 families of two-component type II TA systems: *ccd*, *hicBA*, *hipBA*, *mazEF*, *parD(PemKI)*, *parDE*, *phd-doc*, *relBE* (with 5 subfamilies—*relBE*, *higBA*, *yfeM-yoeB*, *ygiTU(mqsAR)* and *prlF-yhaV*), *vapBC*, *mosAT* and *yeeU.* The less numerous three-component type II TA systems are *ω-ε-ζ*, *pasABC* and *paaR-paaA-parE*. The second classification scheme is based on a set of 44 conserved toxin–antitoxin protein domain pairings and better reflects the versatility and modularity of TA systems [27]. Figure 1 summarizes the variety of type II TA systems identified in bacterial species commonly associated with pathogenesis in humans. Among type II TA system (sub)families, four are most frequently encountered: *vapBC*, *relBE*, *mazEF* and *higBA*, and these account for ~80% of the listed *loci*. (An expanded list of type II TA systems of bacterial pathogens, organized into toxin–antitoxin domain pair groupings, is given in Supplementary Figure S1.)

Due to their widespread occurrence within the accessory genomes of human pathogens, their probable role in pathogenicity and potential as therapeutic targets, type II TA systems have been a subject of growing interest in recent years. Several systematic reviews have focused on the distribution and roles of the TA systems in clinically relevant pathogenic bacteria, including *Escherichia coli*, *Mycobacterium tuberculosis*, *Neisseria gonorrheae*, *Streptococcus* spp., *Burkholderia* spp. and species of the ESKAPE group (*Enterococcus faecium*, *Staphylococcus aureus*, *Klebsiella pneumoniae*, *Acinetobacter baumannii*, *Pseudomonas aeruginosa* and *Enterobacter* spp.) [19,28,29,30,31,32]. An increasing number of studies are being undertaken to describe the repertoire of TA systems in pathogenic bacteria at the level of a given strain or of a whole taxon. Not only do such approaches shed light on the importance of overall TA system networks with respect to the physiology, virulence and evolution of pathogens, but thanks to the large-scale genome comparisons involved, they also lead to the discovery of novel TA *loci*.

*M. tuberculosis* is notable for its abundance of TA systems, especially of the *vapBC*, *mazEF* and *relBE* families (Figure 1), and their activity has been linked to the regulation of adaptive responses to stress caused by interaction with the host and drug treatment [33]. Predictably, *M. tuberculosis* has been the object of studies on the common expression patterns of TA *loci* [34,35,36], as well as cross-activation between homologous systems [37,38], and putative interaction between non-cognate toxin–antitoxin pairs, which remains controversial [39,40,41]. Furthermore, comparative studies of the distribution of TA systems in mycobacteria have demonstrated the variability of TA *loci* between different *M. tuberculosis* lineages, and led to the discovery of putative novel TA systems [42,43]. Similarly, the body of knowledge about the TA systems of *S. aureus* [44] was recently expanded by several studies providing insights into the association between the TA systems landscape and strain phenotypes [45,46,47,48]. A recent in-depth analysis of the diversity and distribution of type II and type IV TA systems in the genomes of *K. pneumoniae* species complex identified several novel toxins and demonstrated the co-occurrence of TA *loci* and clinically relevant genes [49].

Comprehensive strategies for the identification of novel TA *loci* are required in order to gain a greater understanding of the role of TA networks in the biology of bacterial cells. In response to this need, Akarsu and colleagues created the “discovery-oriented” database TASmania in 2019 [50]. Using this newly developed pipeline, they annotated a set of over 41,000 assemblies from the EnsemblBacteria database, resulting in the identification of >2 × 10^6^ candidate TA *loci* [50]. The greater flexibility of TASmania compared to the TAFinder search tool in TADB 2.0 allowed for the identification of a higher number of putative TA *loci*, thus providing a starting point for experimental analyses [50]. Moreover, the “guilt-by-association” strategy used throughout the annotation process, i.e., targeting *loci* directly neighboring orphan toxin or antitoxin genes, facilitated the discovery of new TA protein families. In the case of *Listeria monocytogenes*, TASmania-assisted large-scale genomic comparisons led to the identification of 14 putative TA genes [51].

## 3. Strategies for the Artificial Activation of Toxin–Antitoxin Systems

The growth-inhibitory and lethal consequences of the activity of TA systems led to the proposal that artificial activation of the toxins could provide an effective antibacterial strategy [17,52]. Type II TA systems seem to be most convenient for developing such strategies because many toxins of these systems have been thoroughly characterized and their cellular targets are known [53,54,55]. Moreover, as shown in Figure 1, the presence and conservation of type II TAs have been confirmed in major human-associated bacterial pathogens. Importantly, TA systems have no human homologs, and no pre-existing resistance against TA toxins has been observed. To date, several TA-based antibacterial strategies have been proposed [19,20], but in most cases they are not supported by significant experimental data.

In this review, we focus our attention on the most promising approaches: (i) the direct activation of TA systems by the use of specific molecules that interfere with TAs and imbalance the delicate stoichiometry of active toxin and antitoxin in bacterial cells, and (ii) the indirect activation of TAs by triggering other cellular components whose functions are interconnected with the TA system. Another novel and interesting approach that we consider involves the use of engineered species-specific toxins that can selectively kill selected strains of pathogenic bacteria.

### 3.1. Direct Activation of TA Systems

#### 3.1.1. Disruption or Preventing the Formation of TA Complexes

**Figure 2:**
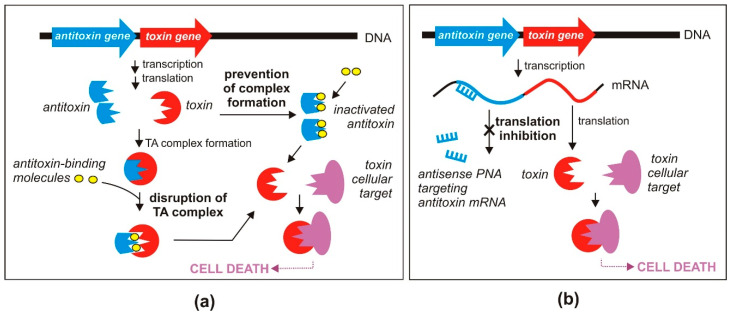
Proposed antibacterial strategies based on the direct activation of toxins of TA systems; (**a**) disruption of the protein toxin and antitoxin complex and/or prevention of protein complex formation; (**b**) inhibition of antitoxin translation (see text for details).

The most straightforward strategy for direct activation of the antibacterial effect of type II TA systems requires disruption of the protein toxin and antitoxin complexes, leading to toxin liberation (Figure 2a). This strategy has been validated by several research groups who focused their efforts on identifying high affinity peptide inhibitors that can efficiently displace toxins from their cognate antitoxins.

Lioy and co-workers selected the ε-ζ TA system from *Streptococcus pyogenes* as their research model because the free ζ toxin was shown to trigger a loss of cell proliferation similar to that caused by known antimicrobials [56]. Using high-throughput methods, they screened several oligopeptide libraries for the ability to impair the assembly of ε–ζ complexes. This led to the identification of a library containing a mixture of 17-amino acid-long oligopeptides that interfered with the TA interaction. However, further subfractionation of this library resulted in a diminished effect. The authors suggested that the disruption of ε–ζ complexes that was originally observed might have been a consequence of the concerted action of several weak-binding oligopeptides. Nevertheless, this study provided a proof-of-concept for the antimicrobial potential of this strategy [56].

A similar approach was applied in the case of two related TA systems—*pemIK* and *moxXT* of *Bacillus anthracis*. The former module encodes the toxin PemK, a ribonuclease whose overexpression exerts a toxic effect in *B. anthracis* cells characterized by the drastic inhibition of protein synthesis [57]. Based on in silico protein structural modeling, several peptides were designed to mimic the *C*-terminal domain of the antitoxin PemI, which is involved in toxin binding. In vitro experiments indicated the effectiveness of the designed molecules and demonstrated the feasibility of disrupting the TA interaction using octapeptides [58]. Similar results were obtained in studies on the related TA system *moxXT*. Based on the crystal structure of the MoxX–MoxT complex, Verma and colleagues [59] designed a series of peptides that effectively disturbed the interaction of MoxT with antitoxin MoxX and stimulated MoxT ribonuclease activity [60].

Analogously, following a detailed analysis of *vapBC* TA systems of *Mycobacterium tuberculosis*, Lee and colleagues [61] designed VapB- and VapC-based peptides that effectively disrupted the TA complexes, causing activation of the VapC toxin ribonuclease [61].

#### 3.1.2. Inhibition of Antitoxin Translation

Toxin release can also be caused by a reduction in the amount of antitoxin molecules in a bacterial cell. One way to achieve such an effect is by blocking antitoxin translation (Figure 2b) [20]. The toxin and antitoxin genes, co-transcribed as a single mRNA, possess separate Shine–Dalgarno sequences [62]. Therefore, inhibition of antitoxin translation should not influence the translation of the toxin protein. This strategy was tested by Równicki and co-workers who designed a peptide nucleic acid (PNA)-based treatment to inhibit translation of the antitoxins of the *mazEF* and *hipBA* TA systems of *E. coli* [63].

The sequence-specific antisense PNAs targeted either *mazE* or *hipB* antitoxin mRNAs and as a result lowered the cellular levels of these transcripts, which caused an effective inhibition of *E. coli* growth [63]. Importantly, the PNA treatment did not change the relative levels of the *mazF* or *hipA* toxin mRNAs. The crucial role of the MazF and HipA toxins in the observed growth inhibition was confirmed by showing that *E. coli* mutants lacking the genes encoding these proteins were “resistant” to treatment with the antitoxin-specific PNAs.

Another PNA-based strategy, described in the same report, used PNA oligomers directed at the cellular target of the toxin, thus bypassing the involvement of the TA system. It was demonstrated that PNAs can mimic the action of the HipA toxin by silencing the *gltX* gene encoding its cellular target, glutamyl-tRNA synthase [63]. For these experiments, the PNA oligomers were conjugated with a cell penetrating peptide—(KFF)_3_K, which indicated the importance of employing an efficient carrier to introduce these antisense oligonucleotides into bacterial cells [63].

The above results confirmed experimentally that TA systems are susceptible to sequence-specific antisense agents and provided a basis for their further exploitation in antimicrobial strategies. Importantly, PNA oligomers exhibit nuclease and protease resistance, high binding affinity to natural nucleic acids, and negligible toxicity to eukaryotic cells [64,65].

### 3.2. Indirect Activation of TA Systems

#### 3.2.1. Enhanced Expression of Proteases Degrading Antitoxins

As previously mentioned, the antitoxins of type II TA systems are more susceptible to degradation by host cytoplasmic proteases than their cognate toxins. In most cases, bacterial Lon protease is involved in antitoxin degradation; however, the two-component protease ClpP, acting in cooperation with the chaperones ClpA or ClpX, is used by some TA systems [66,67,68].

**Figure 3:**
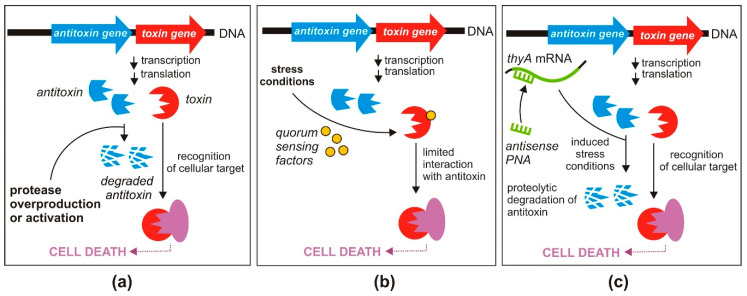
Proposed antibacterial strategies based on the indirect activation of toxins of TA systems: (**a**) activation of the Lon or ClpP proteases that degrade antitoxins; (**b**) triggering TA systems by quorum sensing factors; (**c**) triggering TAs by artificial induction of the stringent response (see text for details).

The depletion of antitoxin molecules results in the liberation of the toxin proteins from TA complexes and relieves transcriptional repression of TA operons, causing increased production of TA transcripts. These events alter the stoichiometry of TA molecules in a cell and ultimately have a lethal effect [69]. Therefore, increasing the level of cellular proteases or designing specific molecules that activate these proteases could be a promising strategy for the indirect activation of toxins (Figure 3a).

Increased protease expression can be achieved by introducing a plasmid carrying a cloned protease gene. Overproduction of the Lon protease is known to be lethal for *E. coli* cells [70]. However, by employing an inducible Lon overproduction system, Christensen and colleagues overcame this problem to demonstrate that the lethality is partially dependent on the *yefM-yoeB* TA system [66]. They showed that overproduction of Lon triggered YoeB-dependent mRNA cleavage, leading to translation inhibition. This, in turn, activated the YoeB toxin by preventing the synthesis of its unstable YefM antidote, which was eventually lethal to the host cells [66].

Proteolysis is a tightly controlled process which can be significantly influenced by specific molecules targeting proteases. For example, acyldepsipeptides (ADEPs) are compounds with antibiotic properties that specifically activate the bacterial protease ClpP. Uncontrolled proteolysis induced in this way inhibits bacterial cell division and results in cell death, possibly with the participation of activated toxins [71].

#### 3.2.2. Triggering of TA Systems by Quorum-Sensing Factors

Another interesting antimicrobial strategy was developed by Kumar and Engelberg-Kulka [72,73]. Their approach targeted *mazEF* TA systems, which are among the most abundant bacterial TA *loci* (Figure 1). Toxin MazF is a sequence-specific endoribonuclease that initiates a programmed cell death pathway in response to environmental stress conditions [74], while MazE is a labile antitoxin that is preferentially degraded by the serine protease ClpAP [14].

The proposed strategy involves the use of a newly discovered group of pentapeptides secreted by bacteria, called extracellular death factors (EDFs), which act in quorum sensing and enhance MazF activity under stressful conditions (Figure 3b) [72]. Interestingly, it was shown that EDFs bind directly to MazF in a sequence-specific manner, and this binding is likely to limit interaction of the toxin with its cognate antitoxin MazE [73,75]. EDFs can also stimulate activation of *mazEF* in heterologous hosts, which might broaden the potential application of this strategy [73,75]. Although an early study on MazF classified its toxicity as lethal to cells [14], this statement has since been revisited, suggesting that MazF is involved in growth arrest rather than cell death [76,77].

#### 3.2.3. Induction of the Stringent Response

Another antibacterial strategy involving the indirect activation of toxins is based on induction of the stringent response, a conserved mechanism that allows bacteria to adapt their metabolism in response to stressful environmental conditions, e.g., nutrient deprivation. Many chromosomal type II TA systems, including the aforementioned *mazEF loci*, are transcriptionally upregulated under stressful conditions [78], and their activity leads to remodeling of cellular metabolism and/or programmed cell death, affecting part of the bacterial community [74].

The stringent response (mediated by the alarmone guanosine 3,5 bispyrophosphate, ppGpp) is activated by different natural starvation and stress signals [79], and this can also be achieved by the application of artificial factors. Równicki and colleagues used sequence-specific PNAs targeting the *thyA* gene of *E. coli*, conjugated with a (KFF)_3_K peptide as a carrier, to trigger MazF toxin production by inducing thymine starvation [63] (Figure 3c). The *thyA* gene encodes thymidylate synthase, an enzyme involved in folic acid metabolism, which normally interferes with *mazEF*-mediated growth inhibition [74]. As shown for *E. coli*, thymine starvation leads to accumulation of ppGpp in bacterial cells, which reduces global transcription [80]. As a consequence, the inhibition of transcription of *mazEF* leads to activation of the MazF toxin [81]. The significantly reduced level of *thyA* mRNA after treatment with a complementary anti-*thyA* PNA and the resulting growth inhibition confirmed the effectiveness of this silencing strategy [63].

### 3.3. Engineered TA Systems in a Targeted Killing Strategy

**Figure 4:**
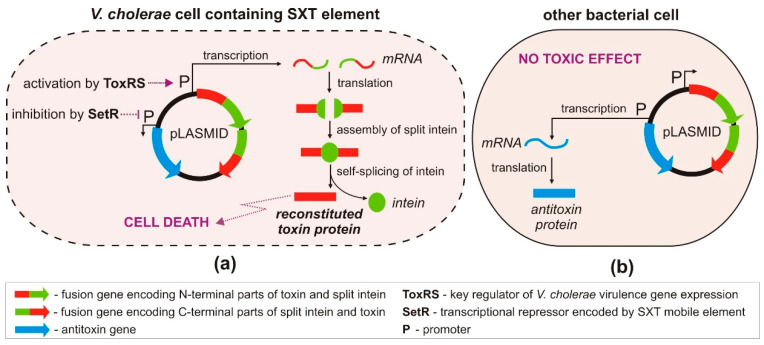
Engineered CcdB toxin as a *V. cholerae*-specific antimicrobial agent. pLASMID—carrier plasmid molecule containing engineered genes of the *ccdAB* TA system. (**a**) In *V. cholerae* cells, by producing ToxRS and SetR transcription factors, transcription of the antitoxin gene is repressed, and expression of engineered toxin genes is activated, leading to cell death; (**b**) in other bacterial cells (lacking both transcription factors), the antitoxin gene is preferentially expressed.

An innovative strategy for the targeted killing of selected pathogenic bacteria, without harming beneficial members of the microbiota inhabiting eukaryotic host organisms, was recently proposed by López-Igual et al. [82] (Figure 4). This strategy is based on the use of the *ccdA-ccdB* type II TA system, encoding the toxin CcdB that poisons DNA gyrase—an enzyme responsible for the negative supercoiling of bacterial DNA [83]. CcdB interferes with the activity of DNA gyrase, inducing it to form a covalent GyrA–DNA complex that cannot be resolved, thus promoting DNA breakage and cell death. This mechanism is closely related to the action of quinolone antibiotics, which also target DNA gyrase [84].

To better control CcdB production in vivo, the gene for this toxin was divided into two parts, and each was fused with DNA encoding split inteins. The generation of a functional toxin occurs in three stages: (i) expression of the two polypeptides, (ii) their association and ligation into a single fusion protein, and (iii) self-splicing of the intein (Figure 4). An important step in this strategy was the construction of a mobilizable plasmid containing genes encoding the antitoxin and the engineered toxin–intein, whose expression could be independently regulated in bacteria harboring specific transcription factors. Although the plasmid could be readily transferred by conjugation from *E. coli* to other bacteria, the toxic effect was observed exclusively in strains of *Vibrio cholerae*. This host specificity was achieved by cloning the engineered *ccdB* toxin–intein genes downstream of a promoter regulated by the transcriptional activator ToxRS, a cholera toxin-associated activator characteristic for *V. cholerae* [85] (Figure 4). Bacterial strains lacking ToxRS were unaffected, including the *E. coli* donor strain and non-pathogenic *Vibrio* spp.

The second component of the TA module, the antitoxin gene *ccdA*, was placed under the transcriptional control of the repressor SetR. The presence of *setR* is considered a hallmark of SXT—an integrative and conjugative mobile element (ICE) of *V. cholerae* that often includes various antibiotic resistance genes [86]. Therefore, expression of the antitoxin gene is repressed in pathogenic multidrug-resistant *V. cholerae* cells containing the *setR* gene, allowing the toxin to poison gyrase and cause a bactericidal effect (Figure 4). However, in cells that lack *setR*, the antitoxin CcdA is produced, neutralizing the effects of the toxin. Thus, the described approach allows species-specific killing of antibiotic-resistant *V. cholerae* strains without affecting the growth of other bacteria present in the mixed populations. The effectiveness of this strategy was confirmed in vivo, in the microbiota of zebrafish and crustacean larvae, where *Vibrio* spp. naturally occur [82].

## 4. Toxicity to Eukaryotic Cells

Although TA *loci* do not occur in eukaryotic genomes, most toxins of TA systems are endoribonucleases that cleave mRNAs irrespective of their origin, be that prokaryotic or eukaryotic. Therefore, before using these toxins in potential antibacterial strategies, it is important to consider possible cytotoxicity and side effects on human cells. Unfortunately, only a few studies have addressed this issue to date. Notably, toxin cytotoxicity was exploited in one study that employed an engineered version of the MazF toxin to reduce solid tumors in mice [87]. Similarly, the VapC toxin from a TA system of *M. tuberculosis* demonstrated pro-apoptotic activity in human cancer cells, regardless of the expression system used. In another study, Chono and colleagues found that the MazF toxin dosage is a critical factor in determining its activity and cytotoxicity in eukaryotic cells [88,89]. The above examples show that mammalian cells are sensitive to the ribonuclease activity of toxins. However, the ability of bacterial TA-system toxins to penetrate eukaryotic cells is currently unknown, and strategies that specifically target pathogens or utilize toxins that lack targets in human cells might prevent any deleterious effects.

## 5. Role of Type II TA Modules in Biofilm Formation and Bacterial Persistence

Two potentially undesirable effects of artificial activation of TAs are bacterial persistence and biofilm formation. Persister cells are a subpopulation of slow-growing or growth-arrested bacterial cells that have a decreased susceptibility to killing by bactericidal antibiotics within an otherwise susceptible clonal population [90]. It has been shown that increased tolerance of biofilms to antibiotics is due to the higher amounts of persister cells within the biofilm community [91,92]. While antibiotics kill the majority of biofilm cells, persisters remain viable and repopulate biofilms when the level of antibiotics drops [93]. Thus, persister cells appear to play a central role in the recalcitrance of chronic and biofilm-related infections [94].

One of the first items of evidence linking type II TA modules to biofilm formation comes from a well-characterized chromosomal TA system *mqsRA* in *E. coli* [95,96]. The *mqsRA* is a unique type II TA system where the toxin gene *msqR* precedes the antitoxin *msqA* [97]. It has been shown that a Tn*5* insertion mutant of the toxin *mqsR* formed less adherent biomass [96]. On the contrary, later reanalysis of the *msqRA* revealed that this TA module does not affect biofilm formation in nutrient-rich conditions [98]. The authors identified two new promoters located in the toxin coding sequence that allow the constitutive expression of *mqsA*, thereby allowing a constant and steady level of the MqsA antitoxin compared to the MqsR toxin. This work, quite understandably, has opened up the debate about the role of TA systems in persistence and biofilm formation [78,99]. This result disproves the role of *mqsRA* (and nine others) TA modules in spontaneous biofilm formation, but does not address the question of a role of TA systems in growth control and biofilm formation under stress conditions. The first persistence-related gene to be identified was *hipA*, encoding the toxin of the *E. coli hipBA* TA system—a serine/threonine kinase that inhibits cell growth by inactivating the glutamyl-tRNA synthetase GltX [100]. This discovery linked TA modules and bacterial persistence for the first time. Since then, correlations between antibiotic persistence and TA systems have been extensively studied. Evidence both for and against the participation of TA systems in persister cell formation has been accumulated, making their contribution to this phenomenon unclear [99,101,102,103,104]. For example, the deletion of 10 ribonuclease-encoding TA systems from the *E. coli* genome was found to decrease the number of persisters. However, it was subsequently shown that this result was influenced by the presence of φ80 bacteriophage contamination [105]. Moreover, reconstruction of this mutant strain demonstrated that deletion of the 10 TA systems did not affect susceptibility to ofloxacin or ampicillin [106]. These contradictory findings have given rise to considerable debate [78,107,108,109], and the relevance of TA systems to bacterial persistence remains unclear.

In contrast, the results of several other studies support the contribution of TA systems to bacterial persistence. For example, overexpression of the toxins RelE or MazF was shown to increase the survival of *E. coli* under antibiotic exposure [9,110]. In another study, the *dinJ/yafQ* TA module was found to be involved in tolerance to cephalosporin and aminoglycoside antibiotics [111]. Further evidence linking type II TA modules and bacterial persistence was obtained in a study on uropathogenic isolates of *E. coli* [112]. TA systems have also been linked to bacterial persistence in *Salmonella*, where the *shpB1* allele, carrying a mutation in the antitoxin of the *shpAB* TA module, was associated with the salmonella high persister phenotype [113]. In addition to the *shpAB* system, 13 other type II TA modules were shown to contribute to persistence in *Salmonella* triggered by stresses encountered during macrophage infection [114]. Furthermore, the overexpression of three acetyltransferase toxins (TacT, TacT2, TacT3—acetylating tRNA) in *Salmonella enterica* was found to increase the level of persisters in the population, and their deletion resulted in a decrease in the proportion of persister cells [115,116]. Since the general mechanism of persistence is still unclear, it is crucial to determine whether the activation of TAs influences persister or biofilm formation before they are considered for use as antimicrobial targets. Besides the type II TA modules, other TA systems have been shown to be involved in persistence in *E. coli*. For further information see important recent publications by Fisher et al. [117], Ronneau and Helaine [78], Wilmaerts et al. [118], Dorr et al. [119], and papers from the Wood group [120,121].

While persistence is among the possible side effects of using TAs as antibacterial targets, these systems can also serve in antipersister strategies [122]. Conlon and colleagues [123] reasoned that a compound capable of striking a target in dormant cells will kill persisters. They used an acyldepsipeptide antibiotic (ADEP4) to globally activate the protease ClpP and showed that this becomes fairly non-specific and kills persisters by degrading over 400 proteins, which forces cells to self-digest. Subsequently, this strategy was used to eradicate a biofilm in an animal model, which confirmed its potential as the basis of therapies to treat chronic infections. In a different approach, Li and co-workers [124] identified a novel inhibitor of the *E. coli* HipA toxin which interfered with persister formation in an antibiotic-independent manner. A comprehensive review describing current antipersister strategies was recently published by Defraine et al. [122].

## 6. Conclusions

Although TA-based antimicrobial strategies have great potential, it is still too early to assess the therapeutic value of toxin activation in clinical settings. Drug discovery and development are time consuming and costly processes, so selecting the right approach will be very important if efforts to produce new antimicrobials based on TA activators are to progress. It is also crucial to select the correct TA targets, and such decisions should be based on data concerning their clinical relevance (i.e., prevalence in antibiotic-resistant clinical isolates), effect exerted on bacterial cells (bactericidal, bacteriostatic), influence on persister cell and biofilm formation, cytotoxicity to human cells and lastly the necessary mode of drug delivery. It would appear advantageous to combine the use of TA activators with conventional antibiotics, since such a strategy could be more effective against a broader spectrum of multidrug-resistant strains [63]. The use of engineered toxins is another promising avenue of research [82,125]. According to the type and properties of such recombinant proteins, they might be applied in targeted antimicrobial strategies or directed against human cancer cells in novel anti-cancer therapies.

## Supplementary Materials

The following is available online at https://www.mdpi.com/2072-6651/12/9/568/s1, Figure S1: The number of the type II TA systems identified in human pathogenic bacteria according to the toxin-antitoxin domain pair system [27] as collected in TADB 2.0 [24].
