# Supplementary material for: Targeting Type II Toxin–Antitoxin Systems as Antibacterial Strategies
Source: Toxins (Basel). 2020 Sep 4;12(9):568. doi: 10.3390/toxins12090568 (PMC7551001; doi:10.3390/toxins12090568)
Supplement: Supplementary file 1 [file toxins-12-00568-s001.pdf]

Toxins **2020**, *12*, 568; doi:10.3390/toxins12090568

Supplementary Materials: Targeting Type II Toxin–Antitoxin Systems as Antibacterial Strategies

Marcin Równicki, Robert Lasek, Joanna Trylska and Dariusz Bartosik

Species	Total	RHH-PIN	Xre-RelE	RHH-RelE	PHD-RelE	Xre-COG2856	PHD-PIN	RHH-MazF	HicB-HicA	Xre-HipA	RHH-GNAT	RHH-COG2929	AbrB-PIN	ArsR-COG3831	HEPN-MNT	AbrB-MazF	Xre-Bro	COG5642-COG5654	YhfG-Fic	Xre-GNAT	COG5606-RelE	AbrB-Fic	MerR-PIN	Xre-Fic	Xre-YgiU	AbrB-RelE	PHD-Fic	COG2442-PIN	Xre-DUF397	Xre-MazF	PHD-MazFn	Xre-PIN	RHH-Fic	XF1863-MazF	Class
Mycobacterium abscessus	8					2								5							1														Actinobacteria
Mycobacterium avium	5	1	1				1							2																					
Mycobacterium bovis	111	64	6	2	6	2	13	4			2		4	2				2					2					2							
Mycobacterium tuberculosis	214	116	12	4	10	4	25	15			5		6	4				4					4					4						1	
Mycobacterium ulcerans	2													1									1												
Corynebacterium diphtheriae	1																																1		
Corynebacterium urealyticum	1					1																													
Nocardia farcinica	16	1	1				1			1				6									1						5						
Propionibacterium acnes	2	1							1																										
Bacteroides fragilis	12		5						1	4										2															Bacteroidia
Porphyromonas gingivalis	2									2																									
Staphylococcus aureus	40				19	9		10								1	1																		Bacilli
Staphylococcus epidermidis	2							2																											
Staphylococcus haemolyticus	3					1		1									1																		
Staphylococcus saprophyticus	2				1												1																		
Streptococcus agalactiae	13			4	1	3		2	1								1							1											
Streptococcus equi	2			2																															
Streptococcus mutans	6			3		1							1			1																			
Streptococcus pneumoniae	34		1	12	5	6			4								2					4													
Streptococcus pyogenes	71			26		24		2	5								11													3					
Streptococcus sanguinis	1																					1													
Streptococcus suis	6			6																															
Bacillus anthracis	8					2		2						2			2																		
Bacillus cereus	12					2		3						3	2		2																		
Bacillus cytotoxicus	5					2		1						1			1																		
Bacillus thuringiensis	3							1						1			1																		
Listeria monocytogenes	3					2		1																											
Enterococcus faecalis	15			2		8									2	2						1													
Clostridium botulinum	25					8		6	1						6		4																		Clostridia
Clostridium difficile	4					2		1																1											
Clostridium perfringens	6					2									1		1			2															
Clostridium tetani	3					2									1																				
Finegoldia magna	4			1		1			2																										
Brucella abortus	5			1		1						1						1				1													Alpha-proteobacteria
Brucella canis	3			1								1										1													
Brucella melitensis	6			2								2										2													
Brucella ovis	2																	1													1				
Brucella suis	4			1								1						1				1													
Ochrobactrum anthropi	14	4	1		1		1		1			2		2								1									1				
Bartonella bacilliformis	4		2										1				1																		
Bartonella henselae	11		4	4					2							1																			
Rickettsia akari	10		1	2	2		1	1	1				2																						
Rickettsia conorii	15		2		1		4	1	1			2	2		2																				
Rickettsia felis	25		2	4	6		4	1	1			2	2		3																				
Rickettsia massiliae	9			1	2		3					1	1		1																				
Rickettsia rickettsii	24		4	2	2		6		2			2	4		2																				
Phenylobacterium zucineum	3					2																									1				
Bordetella bronchiseptica	12	1	3							2		2		2				1							1										Beta-proteobacteria
Bordetella parapertussis	12	1	3						1	3	1	1		1				1																	
Bordetella pertussis	7		1							2				2				1							1										
Bordetella petrii	14					3				4	1	1		2							1					2									
Burkholderia lata	12		3				2			1			2	2				1		1															
Burkholderia mallei	4									2										2															
Burkholderia pseudomallei	14		2	1		1			2	2		1		1				1		3															
Neisseria gonorrhoeae	10	2	2						2		1					2										1									
Neisseria meningitidis	15		3				3					2			1	6																			

(continued on the next page)

(continued from the previous page)

Species	Total	RHH-PIN	Xre-RelE	RHH-RelE	PHD-RelE	Xre-COG2856	PHD-PIN	RHH-MazF	HicB-HicA	Xre-HipA	RHH-GNAT	RHH-COG2929	AbrB-PIN	ArsR-COG3831	HEPN-MNT	AbrB-MazF	Xre-Bro	COG5642-COG5654	YhfG-Fic	Xre-GNAT	COG5606-RelE	AbrB-Fic	MerR-PIN	Xre-Fic	Xre-YgiU	AbrB-RelE	PHD-Fic	COG2442-PIN	Xre-DUF397	Xre-MazF	PHD-MazFn	Xre-PIN	RHH-Fic	XF1863-MazF	Class
Citrobacter koseri	9	1	3	1	1				1	1									1																Gamma-proteobacteria
Cronobacter sakazakii	11		6	1		1				1									1									1							
Escherichia coli	64		20	8	3			7	2	4	4					5			4	1	2					4									
Klebsiella pneumoniae	9	1	2	1						2						1			1								1								
Salmonella enterica	114	5	22	14	6			6		1	22	8	8			1			9		8						4								
Shigella boydii	7		2	1	1						2					1																			
Shigella dysenteriae	9		1	2				1			1					2			1		1														
Shigella flexneri	12		3	2							3					1			3																
Shigella sonnei	7		2	1	1						1					1			1																
Proteus mirabilis	11		3	2	2					2			1										1												
Proteus vulgaris	1		1																																
Providencia rettgeri	1															1																			
Yersinia enterocolitica	14		2	3	1			1		3			2					1									1								
Yersinia pestis	46		17	4	3				7			4						4						3	4										
Yersinia pseudotuberculosis	26		10	2					3			3						3						3	2										
Francisella novicida	1			1																															
Francisella philomiragia	2		1									1																							
Francisella tularensis	6									6																									
Vibrio cholerae	26		4	3	11					1	6	1																							
Vibrio parahaemolyticus	6			3	2					1																									
Vibrio vulnificus	11			3	3				1	3	1																								
Coxiella burnetii	15	1	5		3		2					3			1																				
Legionella pneumophila	22		4	2	2	3			1	6					3								1												
Haemophilus ducreyi	1								1																										
Haemophilus influenzae	32		9	3	2				3	3	1		7		4																				
Acinetobacter baumannii	18		4	2	2				2	1	3	1								3															
Pseudomonas aeruginosa	18			6		3							2							3	1											3			
Aeromonas hydrophila	1					1																													
Stenotrophomonas maltophilia	9		3	1		1				1			1	2																					
Campylobacter concisus	5		3	1					1																										Epsilon-proteobacteria
Campylobacter curvus	3		1		1				1																										
Campylobacter jejuni	4								3	1																									
Arcobacter butzleri	4			1					1						1															1					
Helicobacter pylori	2								2																										
Leptospira borgpetersenii	14	4					2					2		2	2	2																			Spirochaetes
Leptospira interrogans	12	3					2	2					2	1	1	1																			
Treponema denticola	33	7	2	6			2		6	1		6	1		1	1																			
Total:	1463	213	189	155	100	100	72	71	63	61	54	50	49	44	34	30	29	22	21	17	14	12	10	8	8	7	7	6	5	4	3	3	1	1	

Figure S1. The number of the type II TA systems identified in human pathogenic bacteria according to the toxin-antitoxin domain pair system [27] as collected in TADB 2.0 [24]. A number corresponding to an individual species is a sum of the values for all strains of a given taxon. The list was manually curated to represent species frequently associated with infections in humans (including opportunistic pathogens).
